# Defective Kernel 1 (DEK1) is required for three-dimensional growth in *Physcomitrella patens*

**DOI:** 10.1111/nph.12844

**Published:** 2014-05-21

**Authors:** Pierre-François Perroud, Viktor Demko, Wenche Johansen, Robert C Wilson, Odd-Arne Olsen, Ralph S Quatrano

**Affiliations:** 1 Department of Biology, Washington University in St LouisCampus Box 1137, St Louis, MO, 63130-4899, USA; 2 Norwegian University of Life Sciences1432, Ås, Norway; 3 Hedmark University College2318, Hamar, Norway

**Keywords:** cell division, DEK1, meristem, *Physcomitrella patens*, plant morphogenesis, three-dimensional development

## Abstract

- Orientation of cell division is critical for plant morphogenesis. This is evident in the formation and function of meristems and for morphogenetic transitions. Mosses undergo such transitions: from two-dimensional tip-growing filaments (protonema) to the generation of three-dimensional leaf-like structures (gametophores).
- The Defective Kernel 1 (DEK1) protein plays a key role in the perception of and/or response to positional cues that specify the formation and function of the epidermal layer in developing seeds of flowering plants. The moss *Physcomitrella patens* contains the highly conserved *DEK1* gene.
- Using efficient gene targeting, we generated a precise *PpDEK1* deletion (*Δdek1*), which resulted in normal filamentous growth of protonema. Two distinct mutant phenotypes were observed: an excess of buds on the protonema, and abnormal cell divisions in the emerging buds resulting in developmental arrest and the absence of three-dimensional growth. Overexpression of a complete *PpDEK1* cDNA, or the calpain domain of *PpDEK1* alone, successfully complements both phenotypes.
- These results in *P. patens* demonstrate the morphogenetic importance of the DEK1 protein in the control of oriented cell divisions. As it is not for protonema, it will allow dissection of the structure/function relationships of the different domains of DEK1 using gene targeting in null mutant background.

## Introduction

Plant development is mainly driven by positional information, in contrast to animal systems, which to a large extent is dependent on the establishment of cell lineages. Several morphogenetic events in plants require such positional sensing and reception, including embryo pattering, and asymmetric divisions in the root and shoot meristems (see, for a review, De Smet & Beeckman, 2011; Ramsussen *et al.*, 2011). However, it is still unknown how the spatial information required to orient three-dimensional growth is perceived and transmitted in plant development.

Predicted structure, functional analyses, and protein localization establish the Defective Kernel 1 (DEK1) protein as a likely component of three-dimensional sensing in angiosperms. The DEK1 protein predicted structure shows a 21-membrane spanning protein, including a suggested ‘sensor’ loop and a cytosolic calpain cysteine proteinase (CysPc-C2L, or calpain hereafter) as the effector (Lid *et al.*, 2002). The effector cleaves an unidentified substrate protein(s) (Lid *et al.*, 2002, 2005; Margis & Margis-Pinheiro, 2003; Johnson *et al.*, 2005, 2008; Tian *et al.*, 2007) in response to positional activation. Several lines of experimental evidence support a role for DEK1 in epidermis cell specification and maintenance in embryos, as well as for aleurone cells, leaves, and meristems of angiosperms. For instance, in *Arabidopsis thaliana dek1* mutant embryos, divisions after the first asymmetric one fail to occur in the plane specified in wild-type (WT) embryos (Lid *et al.*, 2005). As a consequence, the protoderm (the outermost cell layer of the globular embryo) does not develop normally, and L1-specific markers such as ATML1 and ACR4 are not restricted to their WT patterns (Johnson *et al.*, 2005). Similarly, meristems do not specify the L1 layer, and meristems fail to develop (Johnson *et al.*, 2005; Tian *et al.*, 2007). The weak allele point mutation *dek1-4* of *A. thaliana* specifically abolishes sepal epidermis giant cell formation (Roeder *et al.*, 2012), further confirming that DEK1 plays a key role in epidermis development. Similar results implicating DEK1 in cell fate maintenance have been observed using virus-induced silencing in tobacco (Ahn *et al.*, 2004). Finally, immunolocalization of DEK1 and green fluorescent protein (GFP)-tagged DEK1 studies confirm that DEK1 localizes at the plasma membrane and in endosomal compartments (Tian *et al.*, 2007; Johnson *et al.*, 2008).

Mounting evidence supports an essential role for the DEK1 protein not only in angiosperms, but also in all major land plants, potentially placing this protein at an early stage in the evolution of three-dimensional growth patterns in land plants. This evidence includes the finding that the DEK1 of land plants represents a separate clade of TML-calpains. This clade represents one of four ancestral calpains, which contain large membrane-spanning domains that appear to have emerged 1.5 billion yr ago (Zhao *et al.*, 2012; Liang *et al.*, 2013). Moreover, the catalytic site containing the calpain domain of DEK1 is highly conserved in land plants, from mosses to angiosperms (Liang *et al.*, 2013). Functionally, expression of the endogenous DEK1-calpain domain complements the *A. thaliana dek1-3* null mutant (Johnson *et al.*, 2008; Liang *et al.*, 2013). Similarly, the calpain domain of the moss *Physcomitrella patens* is able to complement the *A. thaliana dek1* mutant (Liang *et al.*, 2013), representing a functional conservation that spans 450 million yr of evolutionary time. By contrast, the calpain domain of the unicellular alga *Mesostigma viride*, an early divergent streptophyte containing a DEK1-like protein, does not complement the *A. thaliana dek1-3* mutant (Liang *et al.*, 2013).

Mosses, including *P. patens*, are extant representatives of one of the earliest divergences of the land plant lineage. They have a relatively simple body plan with much fewer cell types and tissues/organs than in angiosperms. *P. patens*, a bryophyte, forms three-dimensional structures from a single cell twice during its life cycle, once each in the gametophytic (haploid) and sporophytic (diploid) stages. During the sporophytic phase, the single cell zygote develops into an embryo that forms an adult sporophyte with a distinct outer layer epidermis with stomata, which encloses interstitial and meiotic cells. Hence, the sporophytic developmental phase in *P. patens*, although more simplified, is more directly comparable to angiosperm embryo development.

Of particular relevance to assessing DEK1 function in the gametophytic stage, *P. patens* establishes a bud and gametophore, both three-dimensional structures that arise from two-dimensional protonemal filaments (Cove *et al.*, 2006). The key step in bud formation is the establishment of a tetrahedral single cell through spatially coordinated asymmetric cell divisions. This cell acts as a single cell meristem for gametophore development (Harrison *et al.*, 2009). Recently, the protonema-to-gametophore transition, long known to be regulated by cytokinin (Hahn & Bopp, 1968; von Schwartzenberg *et al.*, 2007), has been shown to be directly regulated by AP-2 transcription factor (Aoyama *et al.*, 2012).

In this paper we investigate the role of DEK1 in *P. patens* by creating a deletion mutant in which the single *PpDEK1* gene is removed by homologous recombination. Such *Δdek1* mutants display a complete developmental arrest of the three-dimensional gametophore, as a result of the inability to direct the correct division planes in the emerging bud. In protonema, the effect of the *Δdek1* mutation is milder and growth appears normal, but there is an excess of buds forming compared with the WT. Overexpression of a complete *PpDEK1* cDNA, or expression of the calpain domain alone, successfully complements both phenotypes. Immunolocalization detects the DEK1 protein as punctate granules in the cytoplasm, similar to the location previously shown in angiosperms using the same approach.

## Materials and Methods

### Plant material and growth conditions

All manipulations in this study were performed with the *Physcomitrella patens* Gransden strain. Tissue maintenance and production were routinely performed on BCD medium supplemented with 5 mM ammonium tartrate dibasic (BCDA) as described in Cove *et al*. (2009). *P. patens* tissue and protoplasts were grown under long-day conditions (16 h light (70–80 μmol m^−2^ s^−1^) : 8 h dark). Medium was supplemented with 25 μg l^−1^ Hygromycin B or 30 μg l^−1^ G418 for selection of transformed cells. All phenotypic characterizations were performed on BCD medium unless specifically mentioned (Cove *et al.*, 2009).

To establish the number of buds per filament, a BCD-containing Petri dish was inoculated with 16 equally spaced spot inoculums consisting of 10 μl of protonemal tissue suspension. Bud count was performed after 14 d of growth on one caulonemal filament composed of *c*. 15 cells. Approximately 100 filaments were randomly picked from each plate to establish a budding pattern.

### Nucleic acid extraction and vector cloning

The PCR primers used in this study are listed in the Supporting Information, Table S1. Unless specifically mentioned, all recombinant enzymes were purchased from New England Biolabs (NEB, Ipswich, MA, USA) and were used according to the manufacturer's instructions. Genomic DNA was extracted from *P. patens* protonema using either a quick extraction method (Cove *et al.*, 2009) or the Nucleon Phytopure Plant DNA Extraction Kit (GE Healthcare, Piscataway, NJ, USA). Total RNA was extracted from *P. patens* protonema and/or gametophores using the RNeasy Plant Mini Kit (Quiagen). Subsequent cDNA synthesis was performed with SuperScript® III RT reverse transcriptase using 1 μg total RNA and oligo-dT as primer (Invitrogen).

Sequence from the Pp1s173_19V6.1 (*PpDEK1*) gene model plus 1500 base pairs of 5′ and 3′ extra genomic sequence was used as a template for the design of all primers in this study (see Fig. S1a for map). The deletion vector was designed to remove the full open reading frame (ORF) *PpDEK1* locus. The 5′ and 3′ targeting sequences were PCR-amplified from *P. patens* genomic DNA with primer pairs e*F*/f*R* and g*F*/h*R*, respectively, introducing 5′ *Hin*dIII and 3′ *Xho*I restriction sites and 5′and 3′ *Spe*I restriction sites on the 5′ and 3′ targeting sequences, respectively. The 5′ targeting fragment was then cloned into pBRH (Schaefer *et al.*, 2010) using *Hin*dIII and *Xho*1 restriction enzymes, resulting in plasmid pDelDek1-5′. The 3′ targeting fragment was inserted into pDelDek1-5′ using the *Spe*1 restriction enzyme to generate the pDelDek1 plasmid employed for *P. patens* transformation (Fig. S1b for map). Finally, a suitable open-end DNA fragment for protoplast transformation was obtained by cutting pDelDek1 with *Avr*II and *Kas*I restriction enzyme.

The overexpression complementation vector was designed to express full-length *PpDEK1* cDNA. *PpDEK1* cDNA was amplified using the primers j*F* and k*R*, designed to include the native start codon with the AcV5 short tag at the 3′ end of the cDNA. The amplicon was subsequently cloned into the pCR8/GW/TOPO TA vector (Invitrogen). Sequence orientation and specificity were checked by sequencing, followed by the full-length *PpDEK1* cDNA being transferred into the pT2N2x35-Gateway (Perroud *et al.*, 2011) using the LR Clonase reaction (Invitrogen) to obtain the pT2N2x35-c*PpDEK1* vector (Fig. S1c for map). This expression vector will allow c*PpDEK1* expression under the 2x35S promoter, efficient targeting to the *P. patens* site Phypa_146870 and selection for resistance to the G418 antibiotic. Open-end DNA fragments suitable for protoplast transformation were obtained by PCR amplification using l*R* and m*R* primer pairs designed on each end of the targeting with pT2N2x35::*cDEK1*.

Calpain expression vectors were built as to use the *PpDEK1* genomic locus as regulatory environment. A 3′ targeting sequence 1 kbp long was PCR-amplified from *P. patens* genomic DNA with the primer pairs p*F*/q*R*. The fragments were subcloned into pCR8 GW/TOPO (Invitrogen). The 3′ targeting fragment was then cloned into pBRN (Schaefer *et al.*, 2010) using *Avr*II and *Xho*1 restriction enzymes to generate pTDek1-3′ plasmid. The 5′ targeting sequence and calpain cDNA were subsequently cloned in one step using the In Fusion approach (Clontech Laboratories Inc., Mountain View, CA, USA). The three fragments were prepared as follows: pTDek1-3′ was linearized with the *Xho*1 restriction enzyme. The 1-kbp-long 5′ targeting sequence was PCR-amplified from *P. patens* genomic DNA with the primer pairs r*F*/s*R*. Calpain cDNA sequences from different plants were amplified with the primer pairs t*F*/u*R* from full-length *PpDEK1* cDNA described earlier (*cPpCalp*), the primer pairs v*F*/w*R* from the *A. thaliana* Calpain sequence in plasmid pRPS5A:AtCysPc–C2L–GFP (*cAtCalp*) (Liang *et al.*, 2013), and the primer pair x*F*/y*R* from the *Z. maize* Calpain sequence of the plasmid pRPS5A:ZmCysPc–C2L–GFP (*cZmCalp*) (Liang *et al.*, 2013). Before the cloning reaction, all PCR fragments underwent Enhancer treatment as described by the manufacturer (Clontech). The final vectors were called pTDek1*cPpCalp*, pTDek1*cAtCalp*, and pTDek1*cZmCalp*, respectively (see Fig. S1d–f for a map), and were cut before transformation using the *Avr*II and *Pac*1 restriction enzymes.

### Transformation of *P. patens* and molecular characterization of transformants

*Physcomitrella patens* was transformed using polyethylene glycol (PEG)-mediated transformation as described in Cove *et al*. (2009). Briefly, protoplasts were produced using 7-d-old tissue and submitted to PEG-mediated transformation using the vectors described earlier. Open-end DNA fragments were produced either by double restriction enzyme cut with *Avr*II and *Kas*I with pDeldek1 plasmid or by PCR amplification using l*R* and m*R* primer pairs designed on each end of the targeting with pT2N2x35::*cDEK1*. Protoplasts were regenerated on protoplast regeneration medium (PRMB) overlaid with cellophanes for 7 d. Growing plants were submitted to two selective periods (7 d each), interrupted with one period of selection release (10 d), by transferring cellophanes onto a BCDA medium plate supplemented or not with the appropriate antibiotic. Resistant plants were manually transferred onto fresh medium without cellophane and were grown in order to obtain enough tissue for genotyping and phenotyping.

The Cre recombinase method (Trouiller *et al.*, 2006) was used to remove resistance marker from the primary transformant. Briefly, transient expression of Cre recombinase was performed with the regular PEG-mediated protoplast transformation protocol (Cove *et al.*, 2009) using the *Δdek1* strain, with procedural modifications. Protoplasts were plated on cellophane diluted (25 000 counted protoplasts per 9 cm Petri dish) so as to avoid picking mixed regenerated plant. Protoplast regeneration and test procedures were performed as follows: (1) 4 d protoplast regeneration on PRMB medium; (2) 4 d protoplast growth on BCDA medium; (3) individual plant picking on fresh BCDA plate and growth for 8 d; and (4) replica plating of each individual plant onto BCDA and BCDA+ hygromycin 25 μg l^−1^. Loss of resistance was assessed after 7 d.

Polymerase chain reaction genotyping was performed on DNA extracted using the quick method (see earlier). Southern blot analysis was performed as described in Perroud & Quatrano (2006) using 1 μg of gDNA per digestion. Probes were synthesized with the PCR kit (Roche) according to the manufacturer's instructions. A 5′ fragment was used as a template for the primer pair e*F*/g*R* to make the 5′ probe. A 3′ fragment was used as a template for the primer pair h*F*/i*R* to make the 3′ probe. Finally, gDNA was used as a template for the ee/ff primer pair to make the internal probe.

### Immunohistology

Polyclonal antibodies against *Pp*DEK1 were produced in rabbits by GenScript USA Inc. (Piscataway, NJ, USA). The antibodies were raised against CAVNGTDYVNAREIS peptide and affinity-purified. The manufacturer provided preimmune serum and purified peptide.

*Pp*DEK1 immunolocalization was performed in gametophore tissue from WT and *PpDEK1*_cDNA-ox lines and arrested bud tissue in *Δdek1*. Tissue was fixed in 3.7% formaldehyde prepared in PHEM/dimethyl sulfoxide (DMSO) buffer (50 mM PIPES, 50 mM HEPES, 5 mM EGTA, 1 mM CaCl_2_, pH 6.9, 1% DMSO) overnight at 4°C (Brown & Lemmon, 1995), embedded in Steedman's wax (Vitha *et al.*, 2000). Samples were cut into 10 μm sections using a microtome. Sections were placed on cover slips covered with a thin layer of Meyer's adhesive (Brown & Lemmon, 1995) and, after drying, dewaxed in 100% absolute ethanol. Sections on cover slips were rehydrated in a series of ethanol/PHEM buffer and washed three times in PHEM buffer before blocking. Sections were incubated with block buffer (3% BSA in PHEM) for 1 h at room temperature followed by two washes in PHEM buffer. Primary antibodies were diluted 1 : 250 in 1% BSA/PHEM buffer and sections were incubated in a humid chamber overnight at 4°C. For the controls, antibody-5× excess antigen mixture and 1 : 250 preimmune serum were used. After three washes in PHEM buffer, sections were incubated with 1 : 500 fluorescein isothiocyanate conjugate (FITC) anti-rabbit immunoglobulin G secondary antibodies (Jackson ImmunoResearch Laboratories Inc., West Grove, PA, USA) in a humid chamber for 90 min in the dark at room temperature. The cover slips were washed three times in PHEM buffer and mounted in Prolong Antifade Gold/DAPI (4′,6-diamidino-2-phenylindole) mounting medium (Molecular Probes, Grand Island, NY, USA). Observations were performed 12–48 h after mounting.

### Microscopy

Plants were photographed directly in their growth media without tissue manipulation. Routine observation and low magnification photographs were performed using an Olympus SZX 12 stereomicroscope coupled to a SPOT RT Slider camera driven by SPOT Basic software (Diagnostic Inc., Sterling Heights, MI, USA). Pictures in figures were assembled using Photoshop software (Adobe Systems Inc., San Jose, CA, USA).

To observe buds with confocal microscopy, tissue was stained for 30 min in propidium iodide and washed three times in sterile water before mounting in glass bottom dishes (WillCo Wells B.V., Amsterdam, the Netherlands). Live image acquisition was performed with a Nikon A1 confocal laser microscope system driven by the manufacturer's NIS-Elements software (Nikon Instruments Inc., Melville, NY, USA). All images presented in this study are projection generated using stacking and reconstruction tools of the ImageJ software (Abramoff *et al.*, 2004).

Immunohistological section image acquisition was performed by confocal laser scanning microscopy using a Leica TCS SP5 (Leica Microsystems Inc., Buffalo Grove, IL, USA) setting. Argon 488 nm and UV lasers were used to excite FITC and DAPI fluorochromes, respectively. Detection bandpasses were adjusted to 500–530 nm for FITC and 440–470 nm for DAPI.

## Results

### *DEK1* in *P. patens*

Using the *Zm*DEK1 protein (AAL38187) sequence, a single *PpDEK1* locus (Tian *et al.*, 2007), Pp1s173_19V6.1, is found in the *P. patens* genome (http://www.cosmoss.org), corresponding to the canonical plant *DEK1* gene (*PpDEK1*). The predicted intron–exon pattern for *PpDEK1* is identical to the angiosperm sequence (Lid *et al.*, 2002): 30 coding exons (Fig. S2) and one 5′ untranslated exon. *Pp*DEK1 digital transcript profiling performed across 74 different tissue samples, available at Genevestigator (https://www.genevestigator.com) (Zimmermann *et al.*, 2008), indicated that *PpDEK1* is expressed ubiquitously in the gametophyte (Fig. S3). Reverse transcription polymerase chain reaction (RT-PCR) on total RNA from both protonemal and gametophore tissue of *P. patens* detects transcripts in all tissues examined and confirmed the predicted ORF (*cPpDEK1*). Finally, the deduced protein sequence of *Pp*DEK1 contains the hallmark domains of DEK1: 21 transmembrane domains, a loop insertion between transmembrane spans nine and 10, a nonstructured cytoplasmic arm, and the highly conserved eukaryotic calpain domain (Lid *et al.*, 2002; Zhao *et al.*, 2012; Liang *et al.*, 2013).

### *PpDEK1* ORF deletion

*PpDEK1* ORF deletion was performed by PEG-mediated protoplast transformation (Cove *et al.*, 2009) using the vector pDelDek1 (Fig. S1b). Two independent transformations yielded 85 stable transformants, 28 of them demonstrating a uniform phenotype primary characterized by an absence of developed gametophore. PCR genotyping of the *PpDEK1* locus was performed in two steps. First, we assessed the loss of *PpDEK1* ORF by attempting to PCR-amplify part of the gene targeted for removal using the primer pair a*F* and b*R* (Table S1 for primer sequence). Any detection of the WT size resulted in rejection of the transformant. The 28 transformants without gametophores had all lost the *PpDEK1* ORF by this criterion. Secondly, we PCR-amplified the *PpDEK1* locus using a primer pair c*F* and d*R* designed outside the genomic fragment used to build the pDelDek1 plasmid (Fig. S1a). We set PCR conditions to obtain only a fragment size corresponding to a single replacement. Two of 28 transformants showed a signal corresponding to a single replacement event. Finally, Southern blot was performed to confirm PCR genotyping and ensure a single insertion event in these two candidates. Both selected transformants were confirmed as single copy replacements at the locus (Fig. S4) and showed no sign of secondary nonspecific insertion for both strains (hereafter these strains will be referred to as *Δdek1*).

In order to obtain a mutant without resistance cassette, the Cre recombinase procedure was applied to *Δdek1*. After the procedure, antibiotic-sensitive plants showed an identical phenotype to *Δdek1* and, upon confirmation of loss of resistance by Southern blot analysis, one strain was chosen as the working strain, the *Δdek-cre*.

*Δdek1*and *Δdek-cre* are not lethal, as they allow protonemal tissue to be grown and amplified in a similar manner to the WT. Also, protoplasts produced from 1 wk-old *Δdek1* tissue are viable and are not affected in their regeneration potential; however, several aspects of gametophytic development are strongly affected in both mutant types.

### Effect of *PpDEK1* deletion on gametophore formation

**Fig 1:**
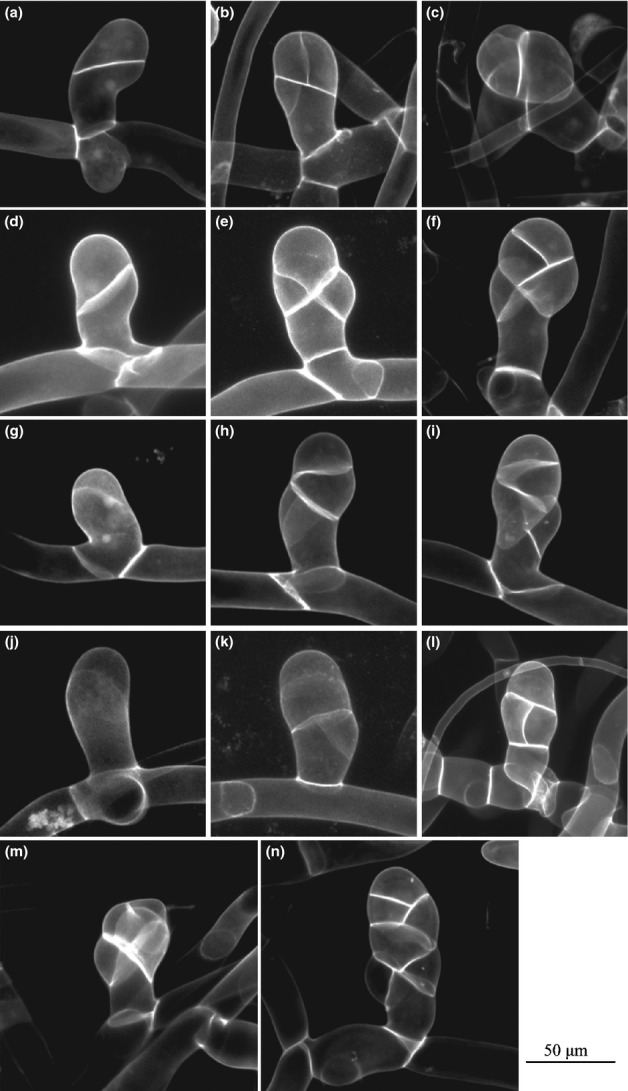
Early bud development is arrested in *Δdek1*. *Physcomitrella patens* filaments containing early stages of bud development were stained with propidium iodide and observed by confocal microscopy. (a–c) Wild-type (WT) bud at the two-cell stage (a), the four-cell stage (b), and the five-cell stage (c). (d–l) Variations in the patterns of cell division in *Δdek1* at the two-cell stage (d, g, j), the four-cell stage (e, h, k), and the five-cell stage (f, i, l). (m) Globular WT bud showing seven cells in a three-dimensional organization. (n) *Δdek1* abortive bud lacking the three-dimensional structure. White chevrons denote end-point projection of cell wall deposition in the four-cell stage.

The most dramatic aspect of the *Δdek1* phenotype is the absence of gametophores. At the two-cell stage of bud formation, which defines the apical and basal domains (Harrison *et al.*, 2009) of the future gametophore, we found no differences between the WT (Fig. 1a) and *Δdek1* (Fig. 1d,g,j) in the plane of the first division. However, the cell division plane of the apical cell was clearly different between the WT (Fig. 1b) and *Δdek1* (Fig. 1e,h,k). Whereas in the WT, the new cell wall intersected the previous cell wall in a clear median position (see Fig. 1b), intersection of the new cell plate in *Δdek1* occurred at random planes (see Fig. 1e,h,k). In addition, the new cell wall in *Δdek1* was curved and wavy, unlike WT. Subsequent apical cell divisions continued to produce the same pattern, with WT divisions forming a tetrahedral cell or a single-cell meristem, which generates future cells in a defined pattern (Harrison *et al.*, 2009), resulting in a three-dimensional structure (Fig. 1c). The corresponding cell division in *Δdek1* was not oriented in the same manner as the WT (Fig. 1f,i,l). This was more clearly shown after subsequent divisions when the WT (Fig. 1m) was compared with the *Δdek1* phenotype, which did not develop to the normal three-dimensional gametophore (Fig. 1n).

### Effect of *PpDEK1* deletion on protonema development

**Fig 2:**
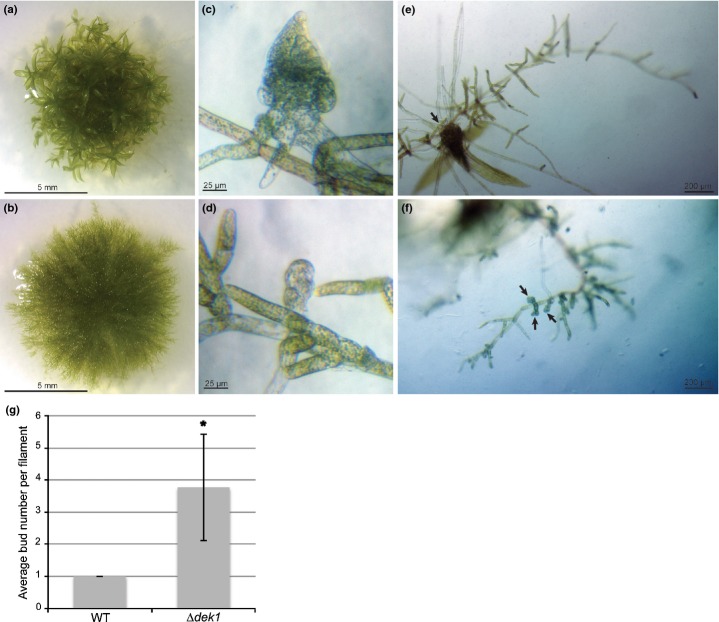
*Δdek1* does not produce gametophores but displays an overbudding phenotype. (a) *Physcomitrella patens* 3-wk-old wild-type (WT) plant showing well developed gametophores; (b) 3-wk-old *Δdek1* plant grown under the same conditions as the WT showing filamentous growth without gametophores; (c) WT early gametophore from 9-d-old culture; (d) *Δdek1* arrested bud from 9-d-old culture; (e) 14-d-old WT filament displaying a single gametophore (arrow); (f) 14-d-old *Δdek1* filament displaying multiple arrested buds (arrows); (g) average number of buds per filament in the WT and *Δdek1* after 14 d. Error bar, ± SD with number of filaments *n *=* *100. Significance of bud average difference between strain assessed using a *t-*test: *, *P *< 10^−10^.

It is clear when comparing Fig. 2(a) (WT) with Fig. 2(b) (*Δdek1*) that the protonema of *Δdek1* did not develop gametophores. However, the absence of a gametophore in *Δdek1* was not a result of a loss of budding capacity, as, when we compared the WT (Fig. 2c,e) with *Δdek1* (Fig. 2d,f), more buds were found on protonemal filaments of *Δdek1*. After 2 wk of growth, *Δdek1* displayed, on average, four buds per 15 filaments, whereas the WT developed only a single bud per 15 filaments (Fig. 2g). A similarly overbudding phenotype can be obtained by exogenous application of cytokinin on the WT filament (Hahn & Bopp, 1968; von Schwartzenberg *et al.*, 2007). To investigate a potential direct effect of *Δdek1* in the signaling pathway for cytokinin-induced bud formation, we treated WT and *Δdek1* filaments lacking buds with 1 μM 6-benzylaminopurine (BAP) for 48 h. Under these conditions, *Δdek1* and the WT both showed identical bud formation patterns (Fig. S5), indicating that the cytokinin pathway itself is not affected in *Δdek1*.

### *cPpDEK1* overexpression complements *Δdek1*

**Fig 3:**
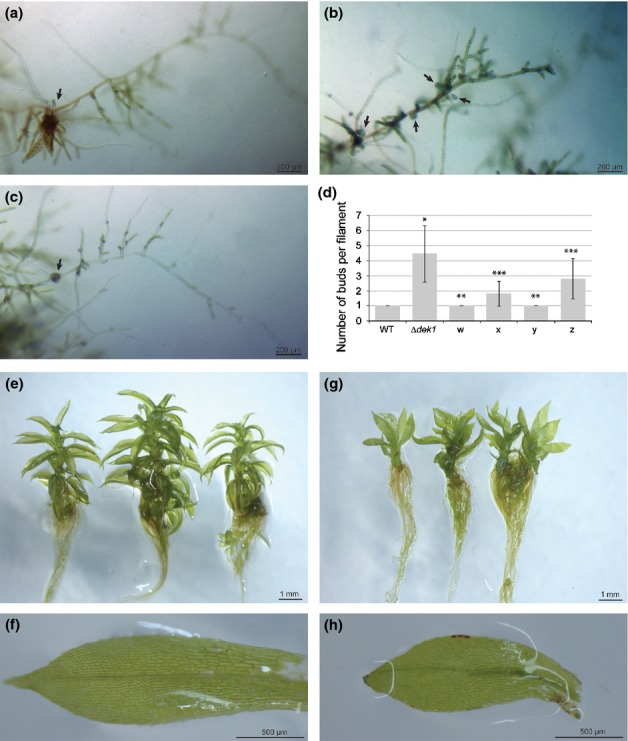
*Δdek1* bud phenotypes are complemented by *PpDEK1* cDNA. (a) *Physcomitrella patens* 14-d-old wild-type (WT) protonemal filament showing a single developing bud (arrow). (b) Fourteen-day-old *Δdek1* protonemal filament showing several abortive buds along the filament (arrows). (c) Fourteen-day-old complemented protonemal filament from strain w showing a single developing bud (arrows). (d) Average number of buds in a 15-cell, 14-d-old filament from six different lines. Error bar, ± SD with number of filaments *n *=* *100. Each complemented line (w, x, y, z) represents an independent transformation of *Δdek1* with *cPpDEK1*. The significance of bud average difference between strain and the WT was assessed using a *t*-test: *, *P *< 1^−10^; significance of bud average difference between the specific strain and *Δdek1* was assessed using a *t*-test: **, *P *<* *1^−10^; significance of bud average difference between strain and both the WT and *Δdek1* was assessed using a *t-*test; ***, *P *<* *1^−6^). (e) Six-week-old WT gametophores grown on BCDA medium. (f) Isolated mature phyllid from the WT gametophore. (g) Six-week-old complemented gametophores grown on BCDA medium. (h) Isolated mature phyllid from a gametophore, which developed from a complemented line w.

*Δdek1* deletion phenotype complementation was achieved by overexpressing *PpDEK1* cDNA using the pT2N2x35-c*PpDEK1* vector (Perroud *et al.*, 2011) (Fig. S1c). *Δdek1* transformation yielded 89 transformed strains showing both WT and mutant phenotypes. The overbudding phenotype was fully complemented in several transformants (compare Fig. 3(c) with Fig. 3a (WT) and Fig. 3b (*Δdek1*)). Among the stable transformants, two independent complemented strains were selected with WT-like bud number (w and y), and two independent complemented strains with intermediary bud number (x and z) (Fig. 3d). They were grown side by side on the same BCD plate. After 2 wk of growth, bud number per filament was reduced compared with *Δdek1* in all four lines (Fig. 3d). In both w and y, gametophore development was delayed by comparison with the WT, but eventually gametophores were formed. As we selected these strains based on the budding number, the phenotypic variation could be attributed to copy number variation of the construct. Also, the use of the 2x35S promoter might not mimic the endogenous expression pattern and could cause the observed variation. Finally, the native transcript contains 29 introns that can play a role in normal transcript stability; hence, expressing a *PpDEK1* cDNA might be different enough from the WT condition to cause the observed phenotype. After 6 wk, WT and *Δdek1* complemented lines displayed differentiated gametophores (Fig. 3e,g) with developed phyllids (Fig. 3f,h). Finally, when all of the complemented lines were subjected to sporulation growth conditions (Perroud *et al.*, 2011), there were no observed morphological defects, but none of the complemented strains formed sporophytes. Additionally, we ruled out a delay effect for the absence of sporophyte by extending the procedure time for 6 months, three times the standard condition, without detecting any sporophyte development.

### The calpain domain of *PpDEK1* alone complements *Δdek1*

**Fig 4:**
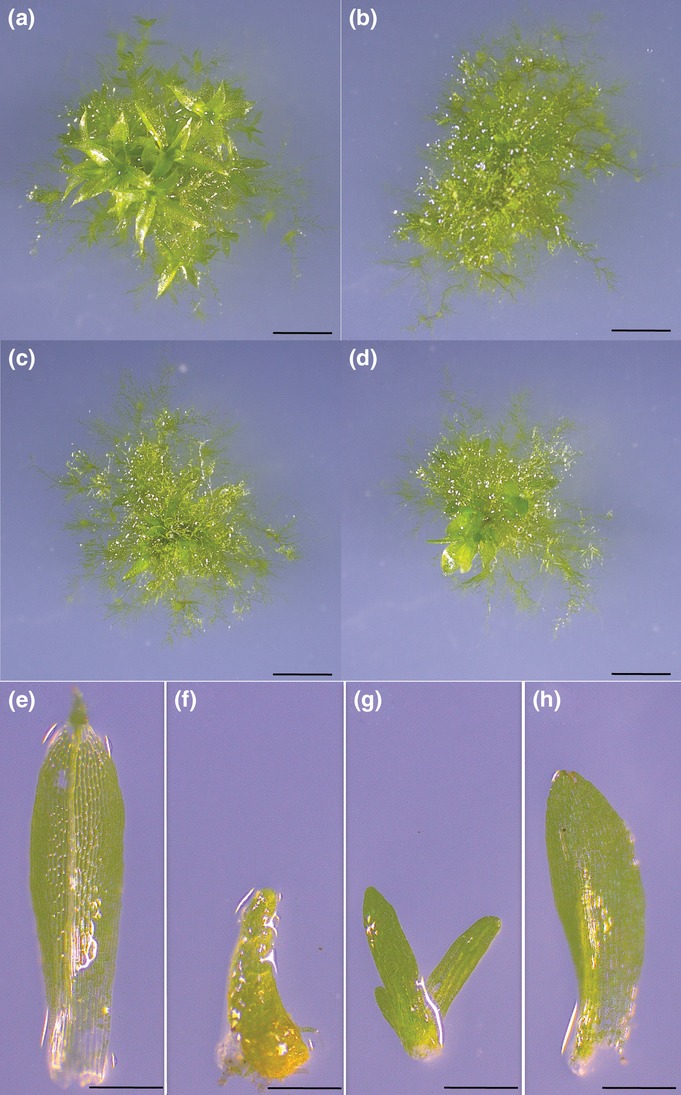
*Δdek1* bud phenotypes are complemented by c*PpCalp*. (a) *Physcomitrella patens* 3-wk-old wild-type (WT) well developed gametophores; (b) complemented strain displaying very small lateral phyllid initials; (c) complemented strain displaying elongated phyllid without lateral widening; (d) complemented strain displaying WT-like phyllid; (e) WT phyllid; (f) phyllid initial from (b); (g) lanceolate phyllid from (c); (h) WT like phyllid from (d) displaying proper elongation and shape (Midrib lateral widening). Bars: 5 mm (a–d); 2.5 mm (e–f).

In Arabidopsis, the endogenous DEK1 calpain domain (c*At*Calp) (Johnson *et al.*, 2008; Liang *et al.*, 2013) and the *P. patens* DEK1 calpain domain (c*Pp*Calp) (Liang *et al.*, 2013) complemented the mutant phenotype. To determine if the calpain domain of *PpDEK1* alone can complement *Δdek1*, we transformed *Δdek1-Cre* with the DEK1 locus-specific vector expressing *cPpCalp* (Tdek1*PpCalp*, Fig. S1d). In most transformants, the *Δdek1-Cre* overbudding phenotype was fully reversed to that of the WT (one bud/15 cells in single caulonemal filaments) and gametophore development was initiated, although gametophore growth was slower in the complemented strains (Fig. 4b–d) than in the WT (Fig. 4a). Additionally, phyllid development displayed varied phenotypes among the independent transformants. After 4 wk growth, some transformants displayed buds with only small phyllid initials (Fig. 4f.). Other transformants showed an elongating phyllid without a midrib (Fig. 4g). Finally, several transformants showed anatomically WT phyllids with margin cells, lateral widening of the phyllid blade and a central midrib (Fig. 4h) comparable to the WT phyllid (Fig. 4e). These phyllid development traits remained uniform in any given transformant and remained unchanged after 3 months of cultivation. Finally, none of the complemented lines formed sporophytes when subjected to sporulation growth conditions (Perroud *et al.*, 2011). We also expressed c*AtCalp* (pTdek1*AtCalp* Fig. S1e) and c*ZmCalpS* (pTdek1*ZmCalp* Fig. S1f) using the same approach. After repeated transformation and generation of > 50 stable transformants, no complementation of either gametophore formation or the overbudding phenotype was observed (data not shown).

### DEK1 cellular localization

**Fig 5:**
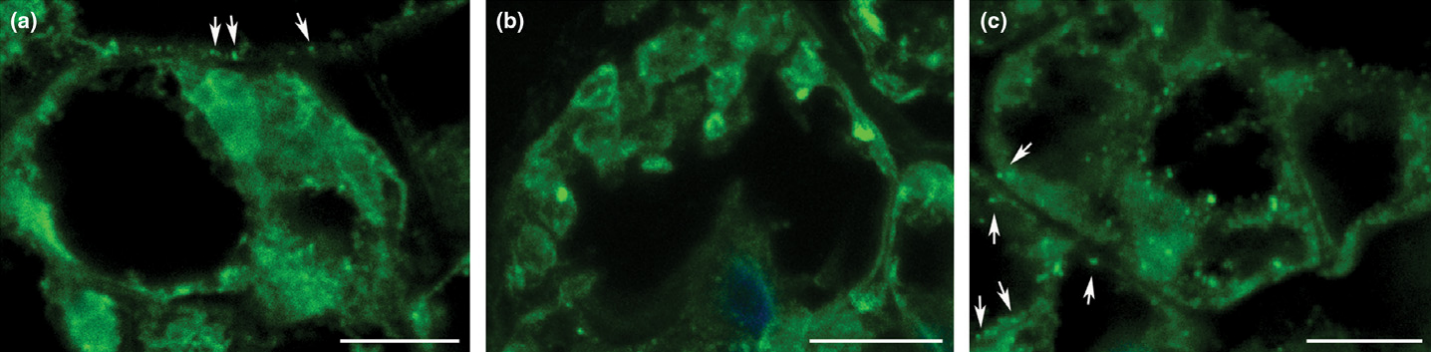
DEK1 *in situ* immunolocalization: (a) *Physcomitrella patens* wild-type (WT) gametophore cells immunostained with PpDEK1 antibody and a secondary antibody conjugated with fluorescein isothiocyanate conjugate (FITC) showing punctate signal (arrows); (b) *Δdek1* bud cell immunostained with PpDEK1 antibody and a secondary antibody conjugated with FITC showing the background without punctate signal; (c) multiple punctuate signals (arrows) in the gametophore cells immunostained with PpDEK1 antibody. Bars, 10 μm.

Tissues from hte WT, *Δdek1* and complemented strain x (Fig. 3) were harvested and processed for immunostaining using the method applied for Arabidopsis DEK1 (Tian *et al.*, 2007). In the WT and complemented strain x (Fig. 5a,c), a punctuated signal was detected that was associated with the cytoplasm retracted from cell walls. The signal was absent in *Δdek1* (Fig. 5b). Additionally, no signal was detected in any strain when preimmune serum was used as the primary antibody or when the antigen peptide was added to the standard procedure (Fig. S6.) The observed punctuated nature and subcellular localization of the DEK1 signal were consistent with and similar to those seen in maize aleurone cells using a similar approach (Tian *et al.*, 2007).

## Discussion

The present study aimed to evaluate DEK1 function in *P. patens* with a focus on the transition to three-dimensional growth derived from a single cell meristem. Using gene targeting (Schaefer, 2002), we generated a full ORF deletion mutant, *Δdek1* (Figs2), which is not lethal in the filamentous stage of the gametophytic tissue. However, two phenotypic effects could be clearly distinguished in the mutant. First, the most dramatic phenotype of *Δdek1* was prevention of the transition from two- to three-dimensional growth, that is, bud development was arrested and gametophores were absent. The main impact of *Δdek1* was in the abnormal early cell division pattern, which led to the absence of the meristematic tetrahedral cell and subsequent gametophore development (Fig. 1). The formation of a single tetrahedral cell in the bud presented strong morphological similarity with respect to inner/outer tissue positioning in angiosperms. Initial cell divisions of the bud in *P. patens* need to be precisely specified to generate a single cell that has one of its faces pointing outward from the apical end of the filament, while the other three faces are in contact with the subtending cell of the bud. Hence, this organization can be viewed as a simplest form of plant tissue with a defined inner/outer structure. There is no analogous single meristematic cell establishment in angiosperms, but the loss of *Pp*DEK1 leading to a failure to establish a tetrahedral cell could reflect a very similar, if not identical, genetic basis to the one observed in angiosperm multicellular meristems. In the developing embryo of *A. thaliana*, the protoderm development in *dek1* mutants is aborted and leads to embryo death (Johnson *et al.*, 2005; Lid *et al.*, 2005). In addition to an embryo that does not fully develop, embryos of the *dek1* null mutants of *Zea mays* lack an identifiable aleurone cell layer, as does cultured endosperm tissue (Becraft *et al.*, 2002; Lid *et al.*, 2002; Gruis *et al.*, 2006). In all these cases, DEK1 has been proposed to play a key role in the inside/outside tissue perception in angiosperm tissue, as evidenced by the null mutant showing aberrant orientations of cell divisions and subsequent developmental arrest. The failure *of Δdek1* to establish the tetrahedral meristematic cell to maintain a polar orientation might use the same genetic ‘subprogram’ that had already been established before the divergence between mosses and the rest of the land plants. Future analysis of the DEK1 defect in *P. patens* in the sporophytic context could confirm these observations in a more comparable developmental phase.

A second clear defect was observed in protonemal tissue. *Δdek1* protonemata initiated more buds than the WT (Fig. 2c–f). This observation is reminiscent of the cytokinin effect on triggering bud formation in protonema. However, we rule out a direct role of DEK1 in cytokinin signal perception and integration because of our observation that the bud induction pattern was identical between the WT and *Δdek1* (Fig. S5). The lack of complex gametophytic tissue development in angiosperms makes it difficult to make comparisons with other land plants.

Complementation experiments using both the full-length *PpDEK1* cDNA (Fig. 3) and the *Pp*Calp calpain domain (Fig. 4) were successful in fully restoring bud number and in allowing gametophores to develop, including the normal appearance of phyllids. Complementation with the *Pp*DEK1calpain motif is analogous to the successful *A. thaliana dek1-3* complementation experiment using *At*Calpain (Johnson *et al.*, 2008; Liang *et al.*, 2013). This strongly suggests a common molecular mechanism behind DEK1 function between mosses and angiosperms, and confirms that DEK1 function lies in the calpain activity and its spatial and temporal regulation. Interestingly, the cross-species complementation that is successful in the moss to angiosperm gene transfer (Liang *et al.*, 2013) is not successful in the reverse direction, as neither *A. thaliana* nor *Z. mays* calpain complements *Δdek1*. An explanation for this observation could lie in the fact that the calpain from tissue in *A. thaliana* and *Z. mays* is from sporophytic tissue. When these calpains are introduced into moss protonema, the calpain from angiosperms cannot function in this gametophytic tissue; perhaps some sporophytic-specific component is required. In either case, some common function and its spatial and temporal regulation reside in the calpain domain of DEK1.

There have been several examples of similar moss/angiosperm comparisons of highly conserved proteins, and several have been tested for functional complementation. The moss *LEAFY* gene failed to complement its counterpart in *A. thaliana* (Maizel *et al.*, 2005), whereas partial complementation was observed with the *P. patens* ABI3 gene in *A. thaliana* (Marella *et al.*, 2006) and with *A. thaliana* Polycomb *FIE* in *P. patens (*Mosquna *et al.*, 2009*)*. Full complementation was observed of the *A. thaliana* RHD6 mutant with its moss homolog (Menand *et al.*, 2007), and the *P. patens* BRICK1 gene can be substituted by its *A. thaliana* homolog (Perroud & Quatrano, 2008). We believe these examples show the opportunities in moss to explore the functional domains in those proteins that are conserved in the green plant lineage.

The protein structure of DEK1, which includes a transmembrane as well as an extracellular and a cytoplasmic domain, fits a postulated model for a membrane protein with these attributes to stabilize an intracellular polar axis that directs the orientation of the cell division plane of the subsequent division (Quatrano & Shaw, 1997). The findings reported here strengthen our proposal that the ancient DEK1 protein was recruited to a novel function in three-dimensional growth regulation in evolving land plants by directing the plane of cell division. In our interpretation, both the ancient nonessential function of DEK1 and the novel function can be observed in *P. patens* in protonemal and gametophore tissues, respectively. In protonema, tip growth is unaffected, whereas the establishment of cell division patterns leading to a three-dimensional bud is aberrant. Understanding moss-specific aspects of *PpDEK1* function may shed light on the origin of DEK1 function and its role in cell and tissue morphogenesis in general. In conclusion, *Δdek1* is opening up the possibility of studying its function in similar functional frameworks across taxa.

## Supporting Information

Fig S1 Locus and construct maps used in this study. **Fig. S2** Exon–intron pattern comparison between chosen land plant *DEK1* genes. **Fig. S3** Digital *PpDEK1* transcript analysis. **Fig. S4** Southern blot analysis of WT and *Δdek1* transformants. **Fig. S5** Exogenous cytokinin treatment effect on WT and *Δdek1* protonemata. **Fig. S6** DEK1 immunolocalization: preimmune serum and peptide competition control. **Table S1** Primers used in this study
