# Supplementary material for: Defective Kernel 1 (DEK1) is required for three-dimensional growth in Physcomitrella patens
Source: New Phytol. 2014 May 21;203(3):794–804. doi: 10.1111/nph.12844 (PMC4285852; doi:10.1111/nph.12844)
Supplement: Fig S1 — Locus and construct maps used in this study. Fig. S2 Exon–intron pattern comparison between chosen land plant DEK1 genes. Fig. S3 Digital PpDEK1 transcript analysis. Fig. S4 Southern blot analysis of WT and Δdek1 transformants. Fig. S5 Exogenous cytokinin treatment effect on WT and Δdek1 protonemata. Fig. S6 DEK1 immunolocalization: preimmune serum and peptide competition control. Table S1 Primers used in this study [file nph0203-0794-sd1.pdf]

Table S1. Primers used in the study.

Name	Sequence (5'-3')
a*F*	TGCATGGACCATAGTCCTGTC
b*R*	AGCTAGAAGAAGCAGGATTGC
c*F*	TTCAATTCCGCATGGCAATTC
d*R*	AAGACGACTACCACTCAGTGC
e*F*	CCTAGGGACCTCCAGACAATACCCAA
f*R*	CTCGAGTTCACATGCTGATACTCCCAG
g*F*	AGATCTTGATGGAGGGATTCTATTTAATAC
h*F*	ATGCATAGAGACGGGAAGAATAAATAG
j*F*	ATGGGGGCGTCCGTGGACGGTG
k*R*	CTAAGACCAGCCGCTCGCATCTTTCCAAGAAAGCGGTTCCAAGATGATAG
l*F*	ATTTAAATGATAAGCCATAGGGTGGCATC
m*R*	ATTTAAATGAAAGAGGTTGGCTGGATTC
n*F*	TGACAGCAAGGGAAGCACGATG
o*F*	ACTATGGACAACACTAGAAGTTC
p*F*	AGATCTTGATGGAGGGATTCTATTTAATAC
q*R*	ATGCATAGAGACGGGAAGAATAAATAG
r*F*	TACGTCGCGACTCGAGGACCTCCAGACAATACCCAATTG
s*R*	AGCCGCTCGCATCTTTCCAAGACATAGTGCCGCTCCCTACTTC
t*F*	AAGATGCGAGCGGCTGGTCTCGCCCCAAAATTGAAACGAAAG
u*R*	ACGAAGTTATCTCGAGCTAAAGCGGTTCCAAGATGATAGA
v*F*	AAGATGCGAGCGGCTGGTCTAGGAAACCCAGGATGGAGACA
w*R*	ACGAAGTTATCTCGAGCTACAAAGCTTCAAGAACAATGGATGC
x*F*	AAGATGCGAGCGGCTGGTCTCCAAGGTTTGAGACACGTGATG
y*R*	ACGAAGTTATCTCGAGCTACCAAACAGCCTCTAGTCTGAT

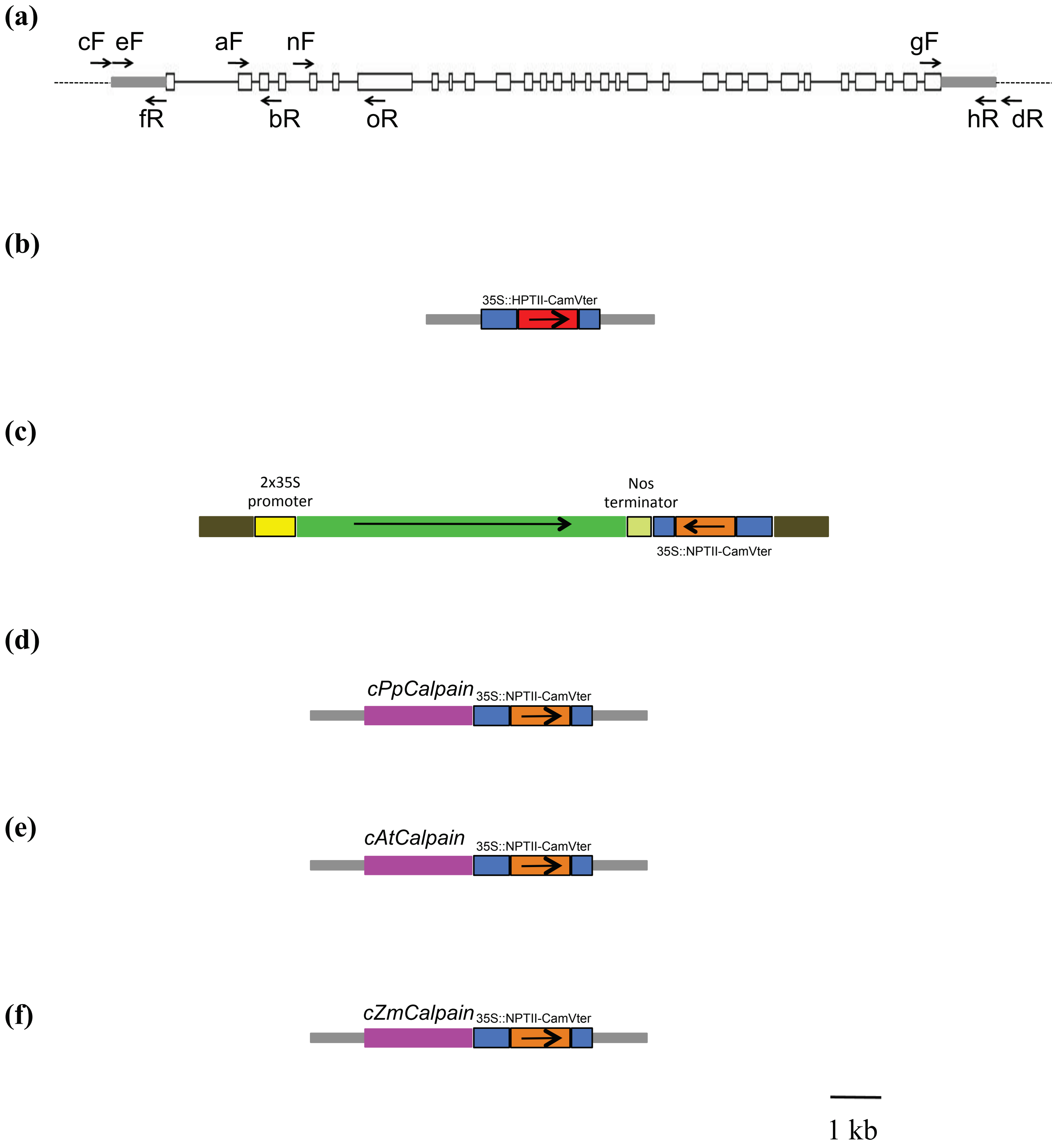
(a)
cF eF
aF
nF
gF
fR
bR
oR
hR dR
(b)
35S::HPTII-CamVter
(c)
2x35S promoter
Nos terminator
35S::NPTII-CamVter
(d)
cPpCalpain
35S::NPTII-CamVter
(e)
cAtCalpain
35S::NPTII-CamVter
(f)
cZmCalpain
35S::NPTII-CamVter
1 kb

Fig. S1.

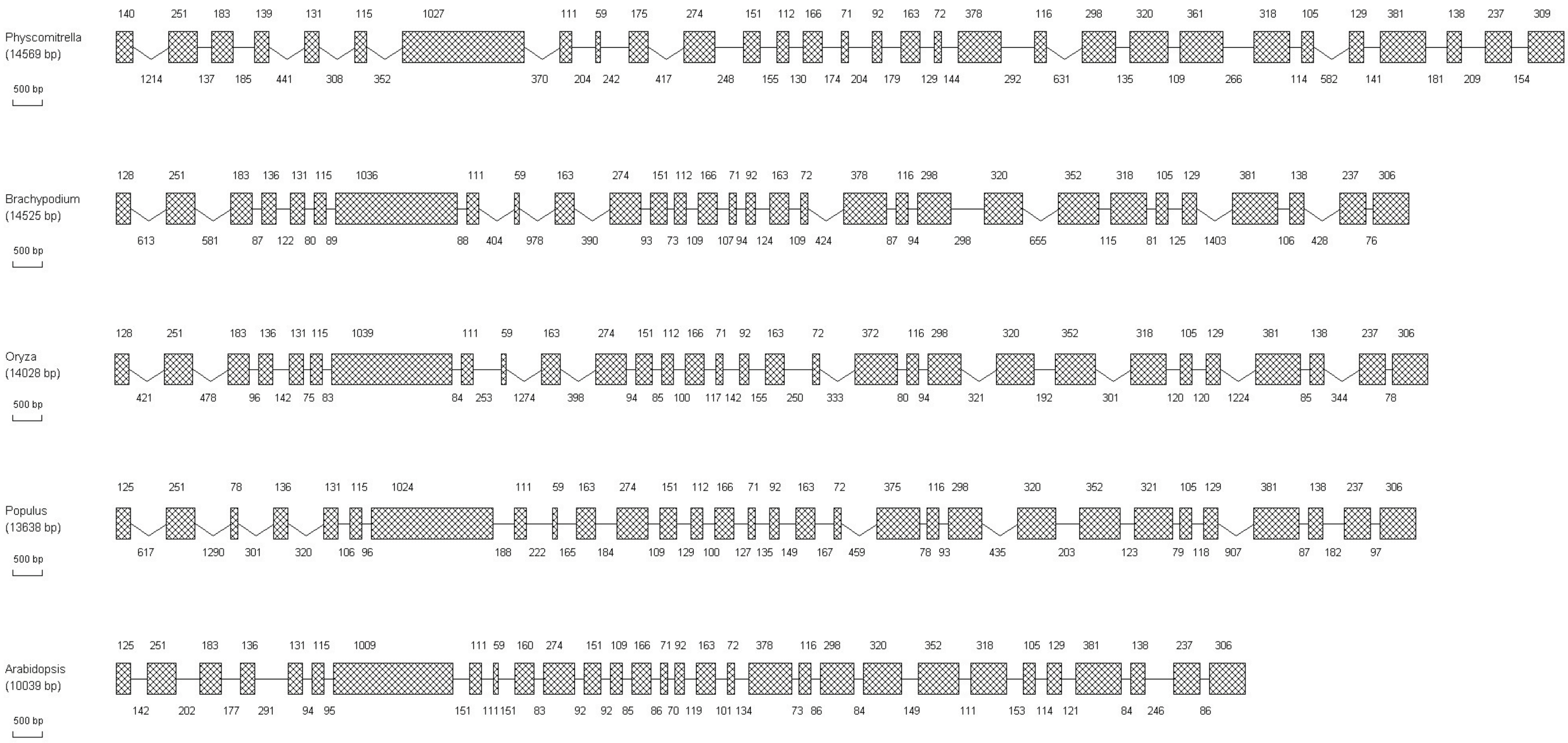
Physcomitrella
(14569 bp)
140 251 183 139 131 115 1027 111 59 175 274 151 112 166 71 92 163 72 378 116 298 320 361 318 105 129 381 138 237 309
1214 137 185 441 308 352 370 204 242 417 248 155 130 174 204 179 129 144 292 631 135 109 266 114 582 141 181 209 154
500 bp
Brachypodium
(14525 bp)
128 251 183 136 131 115 1036 111 59 163 274 151 112 166 71 92 163 72 378 116 298 320 352 318 105 129 381 138 237 306
613 581 87 122 80 89 88 404 978 390 93 73 109 107 94 124 109 424 87 94 298 655 115 81 125 1403 106 428 76
500 bp
Oryza
(14028 bp)
128 251 183 136 131 115 1039 111 59 163 274 151 112 166 71 92 163 72 372 116 298 320 352 318 105 129 381 138 237 306
421 478 96 142 75 83 84 253 1274 398 94 85 100 117 142 155 250 333 80 94 321 192 301 120 120 1224 85 344 78
500 bp
Populus
(13638 bp)
125 251 78 136 131 115 1024 111 59 163 274 151 112 166 71 92 163 72 375 116 298 320 352 321 105 129 381 138 237 306
617 1290 301 320 106 96 188 222 165 184 109 129 100 127 135 149 167 459 78 93 435 203 123 79 118 907 87 182 97
500 bp
Arabidopsis
(10039 bp)
125 251 183 136 131 115 1009 111 59 160 274 151 109 166 71 92 163 72 378 116 298 320 352 318 105 129 381 138 237 306
142 202 177 291 94 95 151 111 151 83 92 92 85 86 70 119 101 134 73 86 84 149 111 153 114 121 84 246 86
500 bp

Fig. S2.

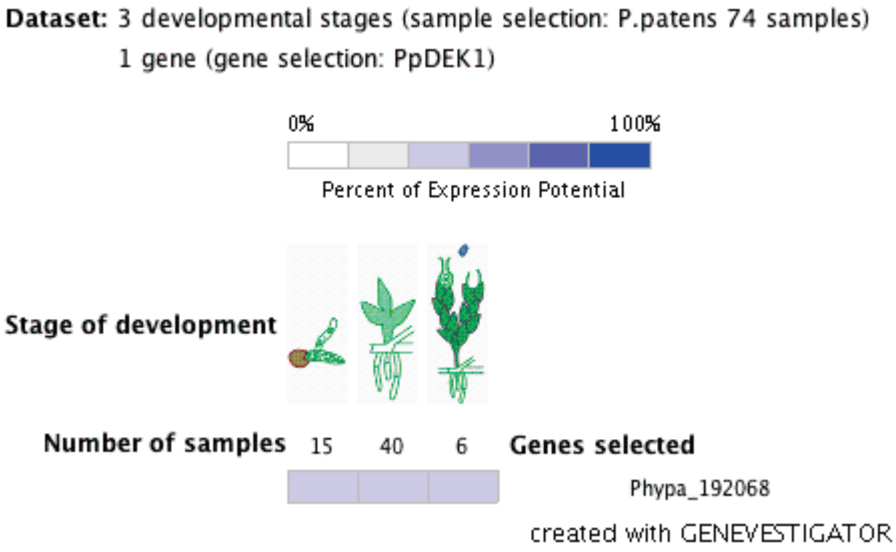
Dataset: 3 developmental stages (sample selection: P.patens 74 samples)
1 gene (gene selection: PpDEK1)
0%
100%
Percent of Expression Potential
Stage of development
Number of samples
15
40
6
Genes selected
Phypa_192068
created with GENEVESTIGATOR

Fig. S3.

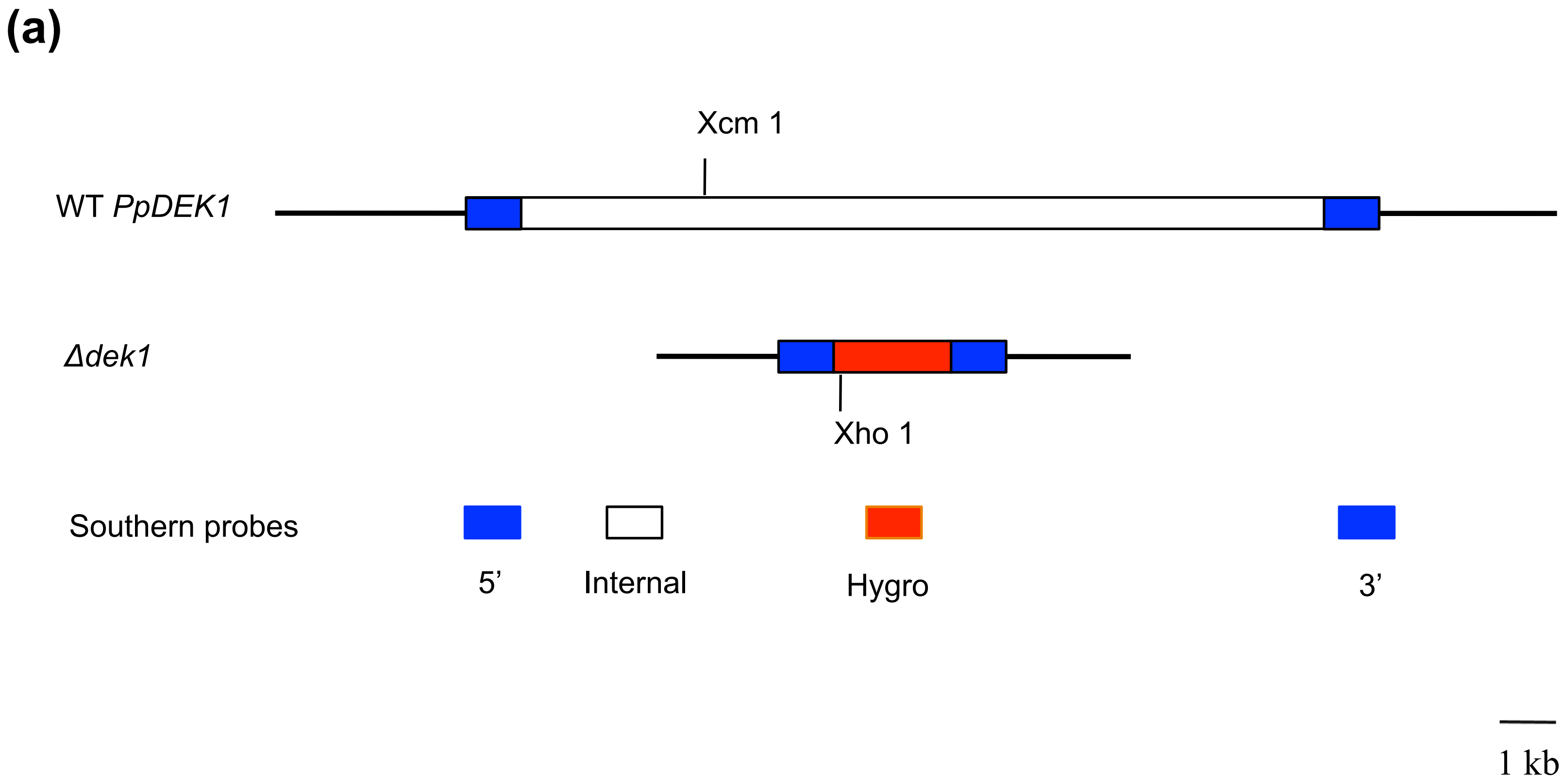
(a)
Xcm 1
WT *PpDEK1*
Δdek1
Xho 1
Southern probes
5'
Internal
Hygro
3'
1 kb

Fig. S4a

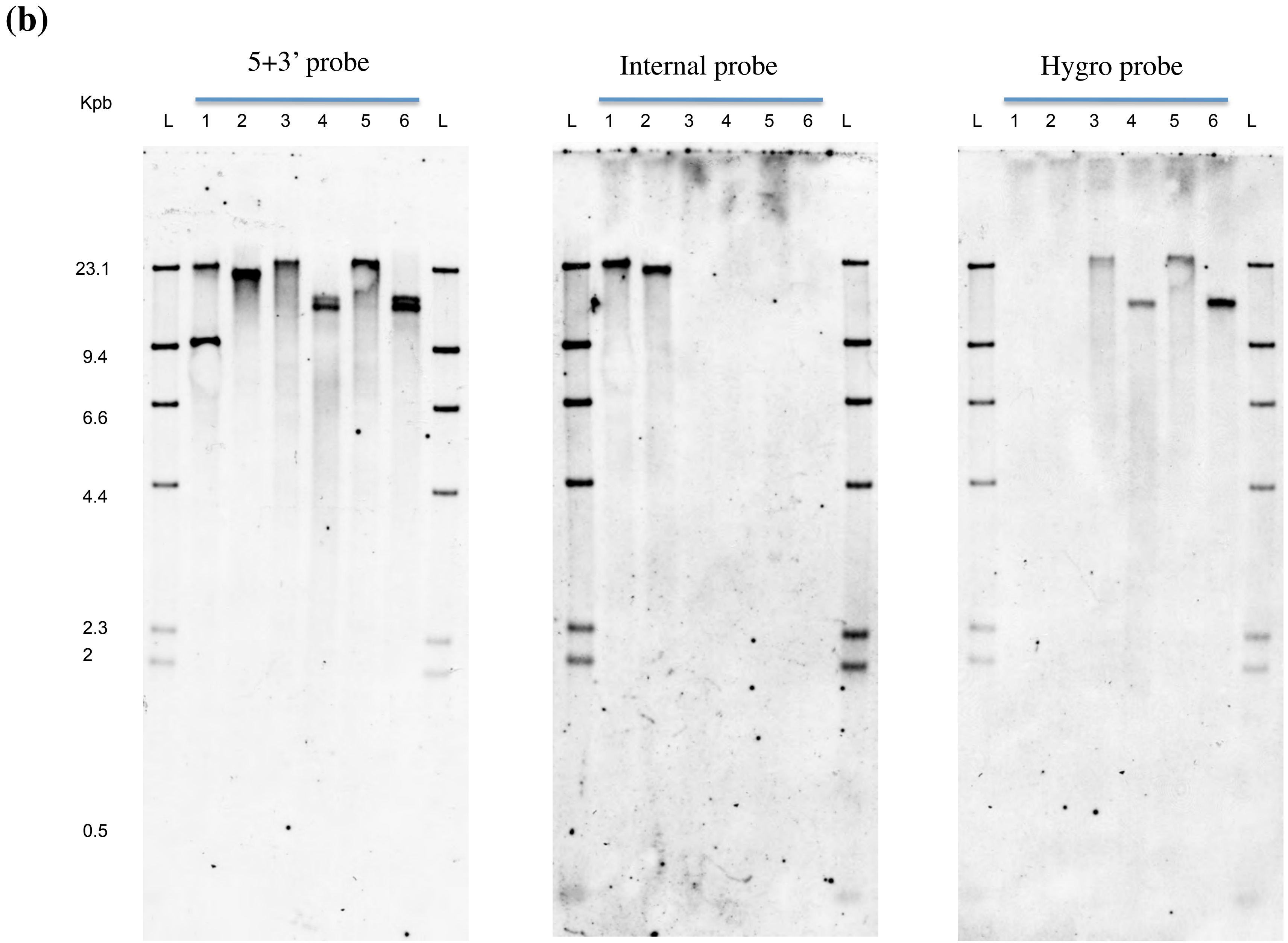
(b)
5+3' probe
Internal probe
Hygro probe
Kpb
L 1 2 3 4 5 6 L
L 1 2 3 4 5 6 L
L 1 2 3 4 5 6 L
23.1
9.4
6.6
4.4
2.3
2
0.5

Fig. S4b

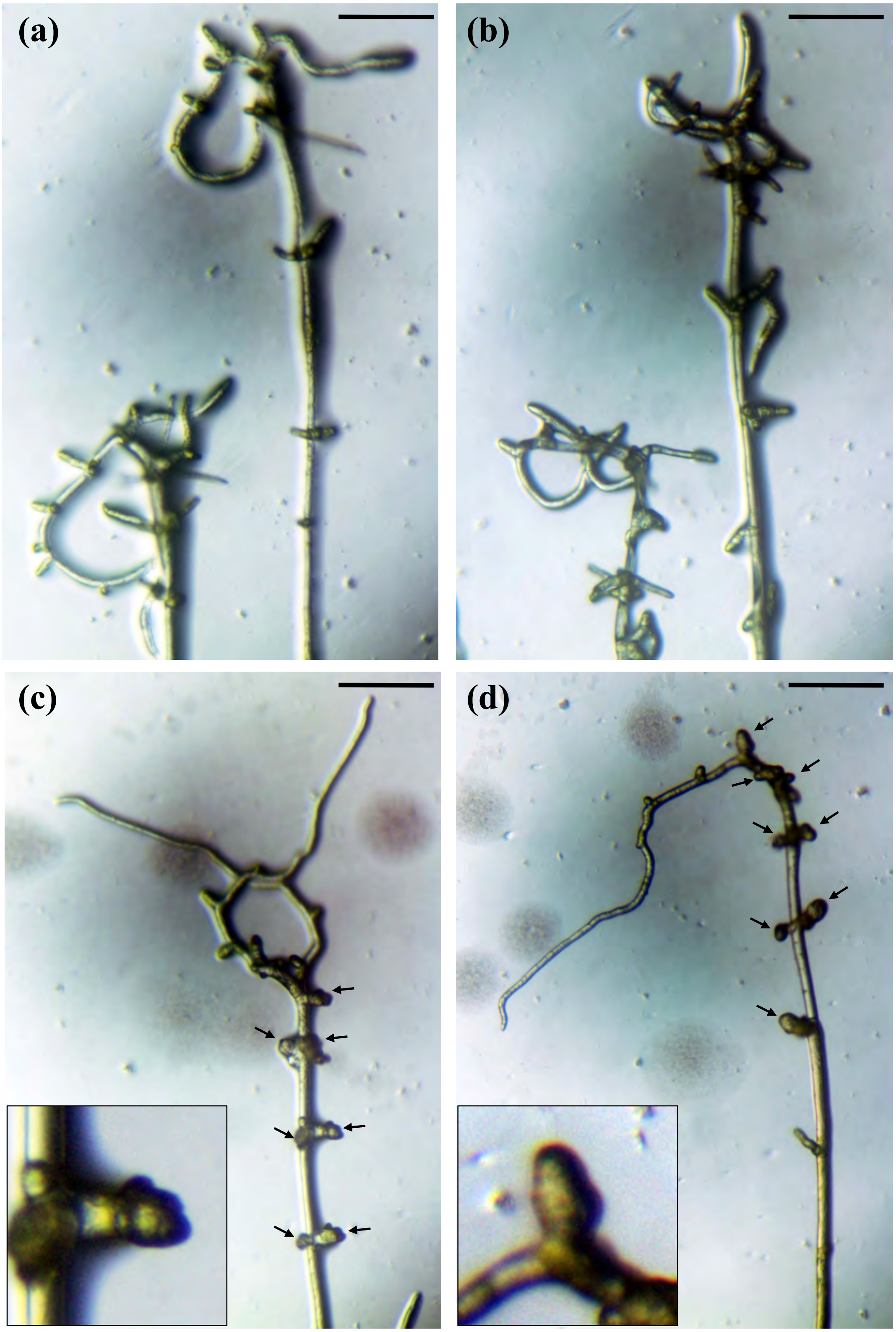

Fig. S5.

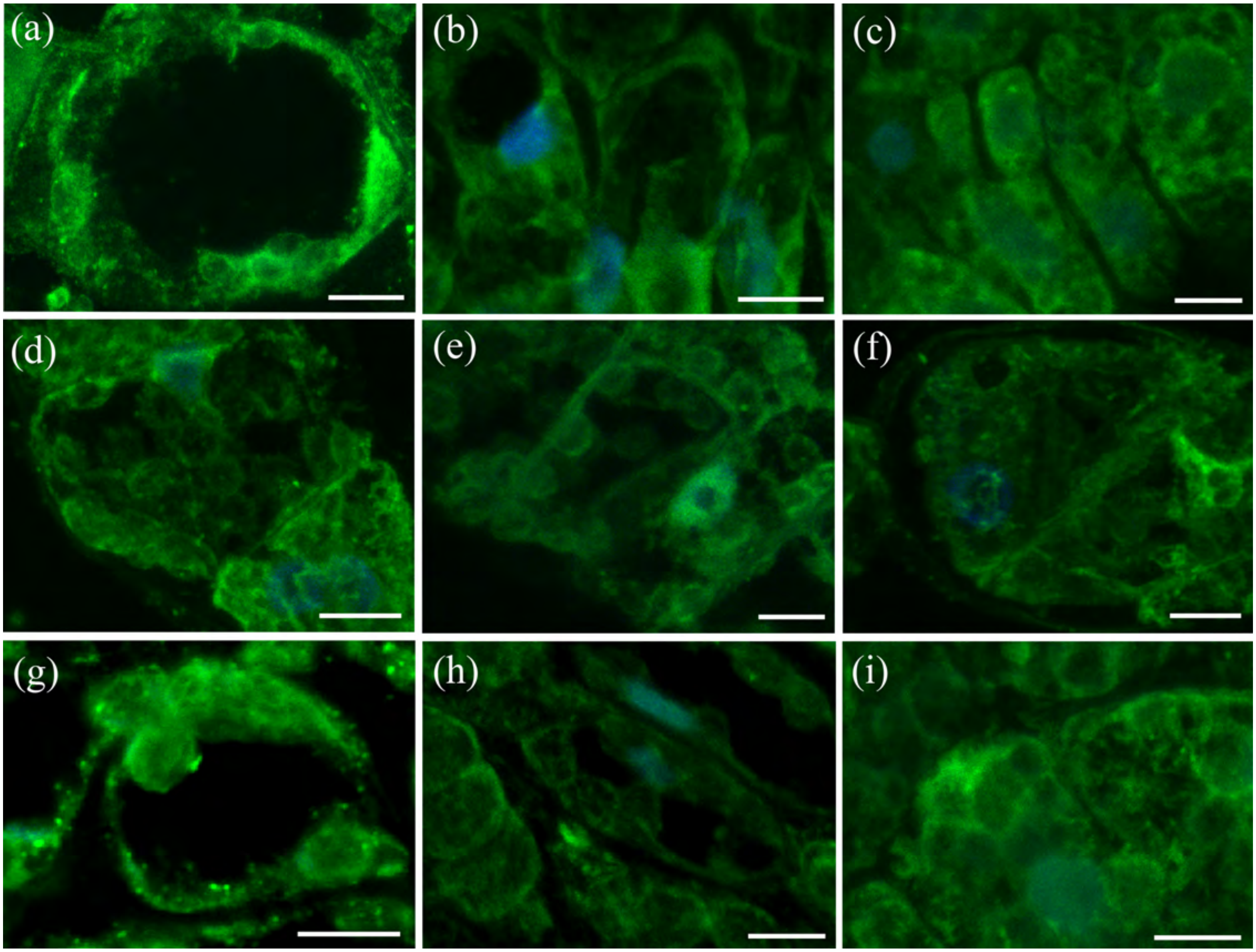

Fig. S6.

Supporting Information Figure Legend.

Figure S1. Schematic of the *PpDEK1* targeted locus and vector maps used in this study. (**a**) The *PpDEK1* locus map (see Table S1 for specific primer sequences). The dashed line indicates the genomic sequence outside the targeted locus. The grey box indicates the targeting sequence, the white box exon, while the black line indicates intron. **(b)** The *PpDEK1* deletion vector (pDelDek1) used to generate Δ*dek1.* The blue and red box represents the hygromycin resistance cassette while the grey box the targeting sequence. Long black arrows show transcription direction of expression cassette. **(c)** Full *PpDEK1* cDNA overexpressing vector pT2N2x35::c*Pp*DEK1used to complement Δ*dek1* strain. The yellow box represents 2x35S promoter, the green box *PpDEK1* cDNA, the light yellow box NopS terminator, blue and orange boxes the G418 resistance cassette while the brown box represents the *P. patens* genomic targeting sequences. **(d-f)** Partial cDNA (calpain coding sequence) expressing vectors targeted to *PpDEK1* locus, respectively pTDek1*cPpCalp*, pTDek1*cAtCalp*, pTDek1*cZmCalp*. The grey box indicates the targeting sequence (identical to **(b)**), the blue and orange box the G418 resistance cassette while the purple box indicates cDNA sequence of **(d)** c*PpCalp*, **(e)** c*At*Calp and **(f)** c*Zm*Calp. All arrows indicate the direction of transcription.

Figure S2. Predicted intron-exon pattern of the open reading frame of the *DEK1* sequence from *P. patens* and selected angiosperms (*Brachypodium dystachion, Oryza sativa, Populus tremula* and *Arabidopsis thaliana).*

Figure S3. Digital *Pp*DEK1 transcript analysis. 74 independent high through put transcript datasets (=samples) have been screened for the presence of *Pp*DEK1 transcript using Genevestigator tool (https://www.genevestigator.com/gv/). Results are expressed on a linear scale.

Figure S4. Southern blot analysis. (**a**) Schematic representation of *PpDEK1* locus before (WT *PpDEK1)* and after transformation (Δdek1) with the restriction enzymes used for the Southern blot analysis. The black line represents the genomic locus outside the targeted

locus, the blue box the sequence used to target the *PpDEK1* locus, the white box the sequence to be deleted, while the red box represents the hygromycin resistance cassette. Bottom row represents the Southern probes used for hybridization. (**b**) Southern blot analysis. 1 µg of genomic DNA for each strain (WT lanes 1 and 2; *Δdek1-1,* lanes 3 and 4; *Δdek1- 2* lane 5 and 6) was digested with the restriction enzymes Xcm1 (lanes 1, 3 and 5) and XhoI (lanes 2, 4 and 6). The gDNA was digested, separated and transferred onto a nylon membrane. The membrane was successively hybridized with three probes; a 5'and 3' probe set (left), the internal probe (middle), and the hygromycin probe (right).

Figure S5. Cytokinin **(**BAP**)** bud induction assay. WT protonemal tissue (**a**) and (**c**); Δ*dek1* protonemal tissue (**b**) and (**d**). Protonemal tissue in (**c**) and (**d**) were treated for 48 hours with 1µM BAP. Both strains showed the same bud initiation pattern upon BAP treatment (**c**) and (**d**) compared to the control without BAP (**a**) and (**b**) (arrows). Black arrow indicates bud. Bar: 200 µm.

Figure S6. DEK1 immunolocalization: pre-immune serum and peptide competition control. **(a)** WT gametophore cells immunostained with PpDEK1 antibody and a secondary antibody conjugated with FITC. **(b)** Control immunostaining of the WT gametophore section with pre-immune serum and a secondary antibody conjugated with FITC. **(c)** Control immunostaining of the WT gametophore section with a mixture of PpDEK1 antibody and antigen peptide. **(d)** Δ*dek1* bud cells immunostained with PpDEK1 antibody and a secondary antibody conjugated with FITC. **(e)** Control immunostaining of the Δ*dek1* bud sections with pre-immune serum and a secondary antibody conjugated with FITC. **(f)** Control immunostaining of the Δ*dek1* bud section with a mixture of PpDEK1 antibody and antigen peptide. **(g)** Immunodetection of the PpDEK1 in the *cPpDEK1*_ox strain using PpDEK1 antibody. **(h)** Control immunostaining of the gametophore sections from *cPpDEK1* overexpression strain using the pre-immune serum and FITC-conjugated secondary antibody **(i)** Control immunostaining of the cells from *cPpDEK1* overexpression strain gametophore using PpDEK1 antibody blocked by the antigen peptide and a secondary antibody conjugated with FITC. White bar: 10 µm.
